# Supplementary material for: Long-Term Results of a Standard Algorithm for Intravenous Port Implantation
Source: J Pers Med. 2021 Apr 24;11(5):344. doi: 10.3390/jpm11050344 (PMC8146737; doi:10.3390/jpm11050344)

Figure S1. Patients disposition diagram

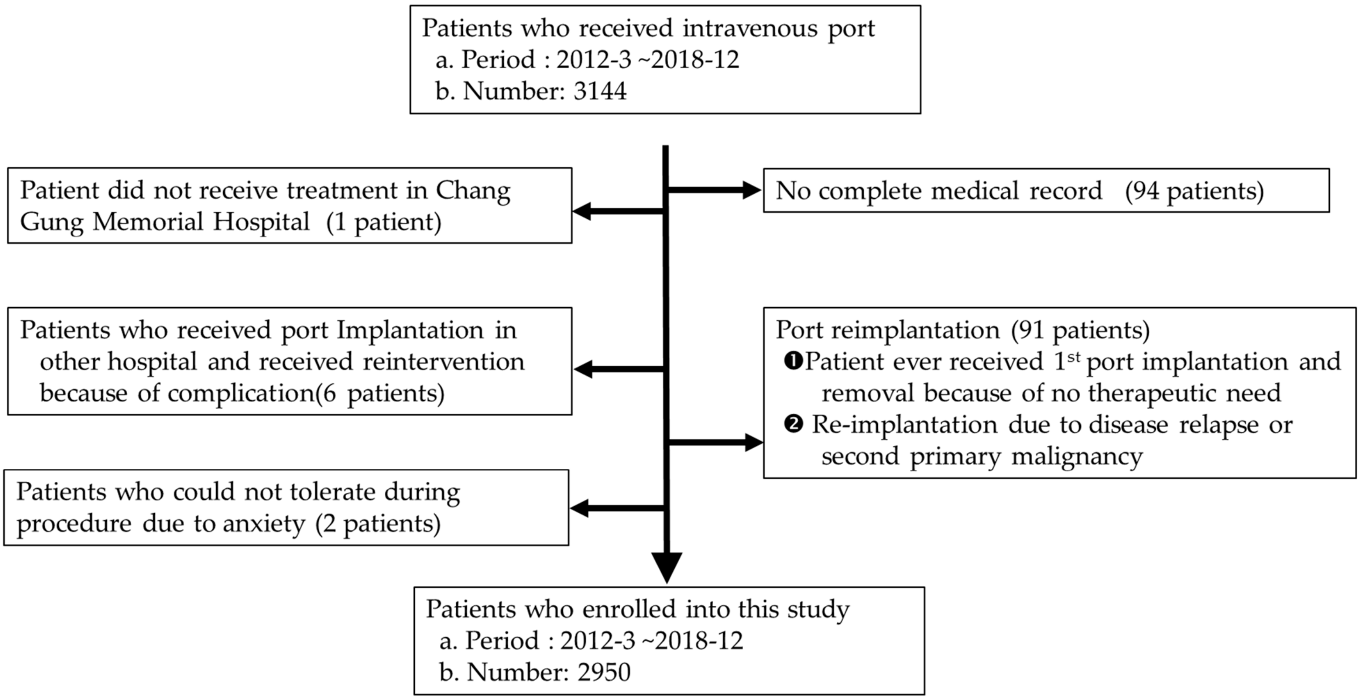

Table S1. Patient characteristics (Whole cohort)

Variables	N(%) or Mean± SD	Variables	N(%) or Mean± SD
Cases number	2950	Operation method	
Gender		Vessel cutdown	1,870 (63.4%)
Male	1713 (58.1%)	Wire assistance without puncture	602 (20.4%)
Female	1237 (41.9%)	Wire assistance with puncture	353 (11.8%)
Age (years)	58.6 ± 12.8	Wire and venogram assistance	
Body height (Kg)	161.2 ± 8.9	a. Without puncture	13 (0.4%)
Body weight (cm)	60.8 ± 12.0	b. Puncture	37 (1.2%)
Body mass index (BMI)	23.3 ± 3.9	Echo guide puncture	75 (2.5%)
Underlying malignancy[1]		Operation time (Minute)	
Head and neck	302 (10.2%)	Vessel cutdown	27.3± 10.8
Thorax	1,274 (43.2%)	Wire assistance without puncture	30.1± 10.5
Abdomen	1,133 (38.4%)	Wire assistance with puncture	40.3± 15.8
Pelvis	13 (0.4%)	Wire and venogram assistance	
Soft tissue	17 (0.6%)	a. Without puncture	33.2± 9.7
Hematology	250 (8.5%)	b. Puncture	48.6± 16.5
Other	21 (0.7%)	Echo guide puncture	61.0± 18.2
Side		Post-operation quality	
Right	2,640 (89.5%)	Catheter-nut angle (°)	169.8± 7.5
Left	310 (10.5%)	Tip location (cm)	1.2± 1.5
Entry vessel		Port type	
Superior vena cava route	2,905 (98.5%)	B'Braun Fr. 6.5	859 (29.1%)
Cephalic vein	2,501 (84.8%)	Bard X port Fr.6 /Fr.8	765 (25.9%)
Thoracoacromial vein deltoid br.	326 (11.0%)	Bard power port Fr.6	932 (31.6%)
Internal jugular vein	75 (2.5%)	Polysite Fr.7	394 (13.4%)
Other[2]	3 (0.10%)	Functional period (day)	542.6 ± 497.2
Inferior vena cava	45 (1.5%)	Follow-up status	
Greater saphenous vein	45 (1.5%)	Alive	1,813 (61.5%)
		Expire	768 (26.0%)
		Against advise discharge	369 (12.5%)

[1] 43 male patients have diagnosed double cancers , 17 female patients have diagnosed double cancers

[2] Other: 38 right greater saphenous veins, 7 left greater saphenous veins, 1 right axillary vein, 2 right external jugular veins

Table S2 Complication rates and incidence between Year 2005[7] and Year 2018

Vessel / Years / No / Complication *Rate*	Cephalic vein		Subclavian vein		Thoracoacromial vein		Internal jugular vein		Other*		Total	
Years	2005[7]	2018	2005[7]	2018	2005[7]	2018	2005[7]	2018	2005[7]	2018	2005[7]	2018
No	1216	2501	234	0	0	326	63	75	29	48	1542	2950
Pneumothorax	0%	0%	0.85%	0%	0%	0%	0%	0%	0%	0%	0.13%	0%
Hematoma	0.41%	0%	0%	0%	0%	0%	0%	0%	0%	0%	0.32%	0%
Port rotation	0.25%	0.04%	0%	0%	0%	0%	0%	1.33%	0%	0%	0.19%	0.07%
Catheter kinking	0.58%	0%	0.43%	0%	0%	0%	0%	0%	0%	0%	0.45%	0%
Fracture	2.38%	0%	12.39%	0%	0%	0%	1.59%	0%	0%	0%	3.83%	0%
Migration	2.30%	0.64%	2.14%	0%	0%	0.92%	0%	0%	3.45%	0%	2.2%	0.64%
Malfunction	3.21%	0.32%	2.99%	0%	0%	0.61%	0%	2.67%	6.90%	4.17%	2.98%	0.47%
Infection	9.04%	1.28%	6.41%	0%	0%	1.23%	7.94%	1.33%	13.79%	2.08%	8.69%	1.29%
Pocket erosion	1.15%	0%	0%	0%	0%	0%	0%	0%	3.45%	0%	0.97%	0%
Deep vein thrombosis	1.15%	0.56%	0%	0%	0%	0.61%	1.59%	0%	0%	0%	1.17%	0.51%
Pain	0.16%	0%	0%	0%	0%	0%	0%	0%	0%	0%	0.13%	0%

Vessel / Year / No / Complication *Incidence*	Cephalic vein		Subclavian vein		Thoracoacromial vein		Internal jugular vein		Other		Total	
Year	2005[7]	2018	2005[7]	2018	2005[7]	2018	2005[7]	2018	2005[7]	2018	2005[7]	2018
No	1216	2501	234	0	0	326	63	75	29	48	1542	2950
Pneumothorax	0	0	0.0221	0	0	0	0	0	0	0	0.003	0
Hematoma	0.0100	0	0	0	0	0	0	0	0	0	0.0096	0
Port rotation	0.0060	0.0007	0	0	0	0	0	0.0213	0	0	0.0048	0.0012
Catheter kinking	0.0140	0	0.0110	0	0	0	0	0	0	0	0.0127	0
Fracture	0.0579	0	0.3204	0	0	0	0.0397	0	0	0	0.0948	0
Migration	0.0559	0.0117	0.0552	0	0	0.0177	0	0	0.1414	0	0.0546	0.0119
Malfunction	0.0779	0.0058	0.0773	0	0	0.0118	0	0.0426	0.2828	0.1274	0.0772	0.0087
Infection	0.2197	0.0234	0.1657	0	0	0.0237	0.1986	0.0213	0.5657	0.0637	0.2133	0.0237
Pocket erosion	0.0280	0	0	0	0	0	0	0	0.1414	0	0.0241	0
Deep vein thrombosis	0.0280	0.0102	0.0331	0	0	0.0059	0.0397	0	0	0	0.0289	0.0093
Pain	0.0020	0	0	0	0	0	0	0	0	0	0.0030	0

* Definition of Other entry vessel

2005: Other vessel including: 13 right greater saphenous veins, 5 left greater saphenous veins, 7 Deltoid branch of thoracoacromial vein, 1 right axillary vein, 1 left axillary vein, 1 right external jugular vein and 1 left external jugular vein

2018: Other vessel including: 38 right greater saphenous veins, 7 left greater saphenous veins, 1 right axillary vein, 2 right external jugular vein

Figure S2 Wire assistance with venography to overcome small vessel caliber

A. Small caliber of deltoid branch of thoracoacormial vein. Intraoperative venogram via 20 Gauze IC needle and showed tiny venous drainage (black arrow) to subclavian vein with 2 sharp turns (white arrow).
B. Cannulated with 0.018 V-18 inch control via 20 Gauze IV needle and pass the first turn (white arrow).
C. Cannulated with 0.018 inch V-18 control via 20 Gauze IV needle and pass the second turn (white arrow).
D. 0.018 V-18 wire pass to subclavian vein.
E. Smoothen configuration with rigidity of 0.018 inch V-18 control wire.
F. Adjust metallic wire pass from subclavian vein to superior vena cava.
G. Peel apart sheath was utilized for tunnel creation via metallic wire.
H. Post-operation chest X-ray after port implantation.

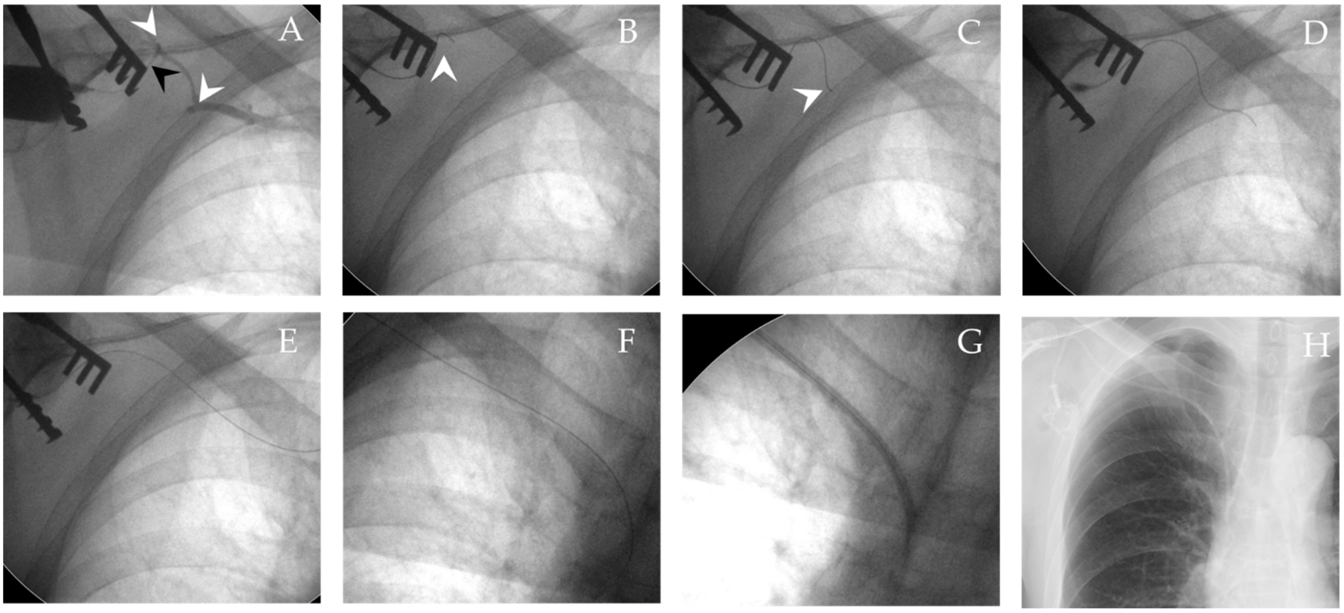

Figure S3 Wire assistance with venography to overcome sharp angle at confluence site between cephalic and subclavian vein

A. Catheter stacked at junction site between cephalic vein and subclavian vein.
B. Intraoperative venography was done via catheter and revealed sharp angle between cephalic and subclavian vein (white arrow).
C. Cannulate with 0.035 Terumo wire via catheter. Catheter advance to junction site to re-cannulate wire to superior vena cava. However, repeat to right IJV and repeat intraoperative venogram was done.
D. Intraoperative venogram showed near 90 degree turn at junction site between subclavian vein and superior vena cava. Cannulate with 0.035 Terumo wire under road mapping guidance.
E. Catheter was implanted over the wire under fluoroscopy.
F. Postoperative chest X-ray after port implantation.

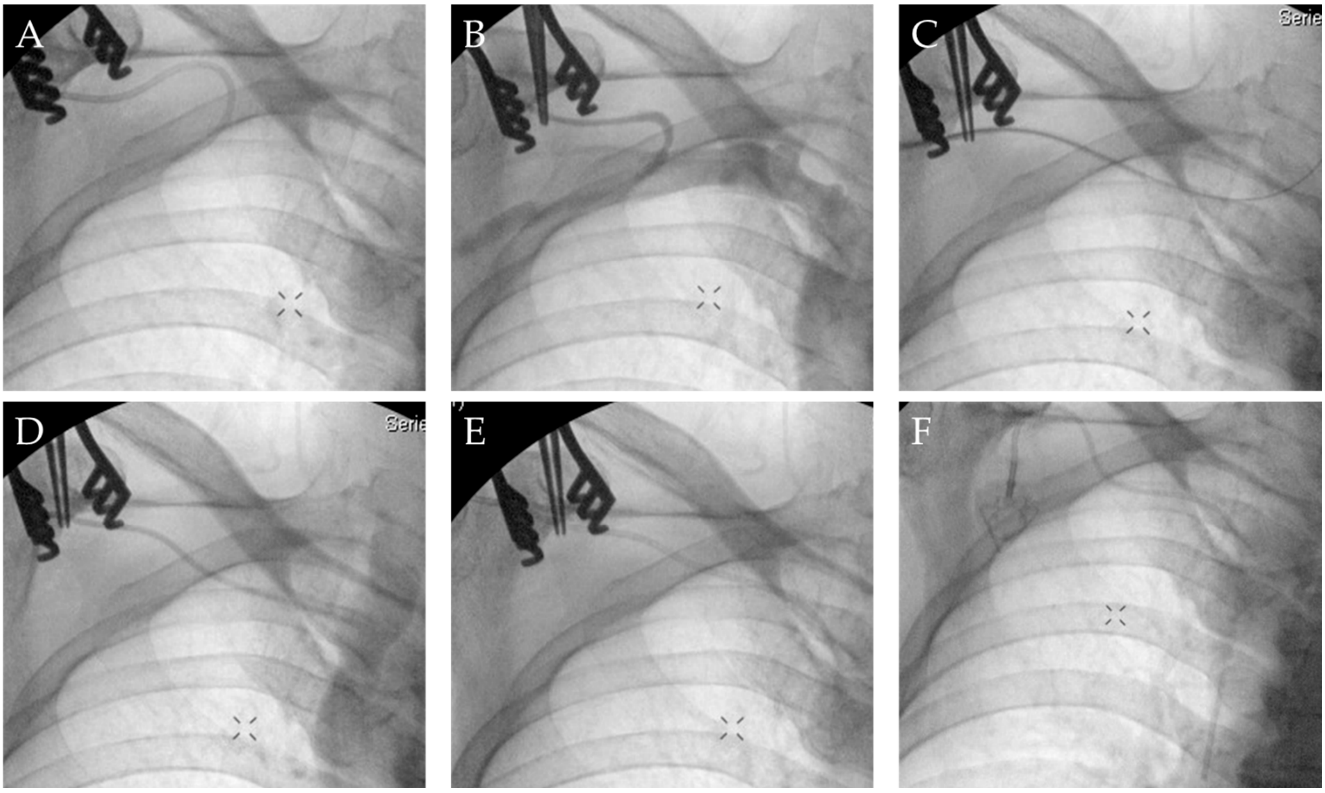

Supplement: Supplementary file 1 [file jpm-11-00344-s001.zip › jpm-1159587-supplementary.pdf]
